# Human Mesenchymal Stromal Cell-Derived Exosomes Promote In Vitro Wound Healing by Modulating the Biological Properties of Skin Keratinocytes and Fibroblasts and Stimulating Angiogenesis

**DOI:** 10.3390/ijms22126239

**Published:** 2021-06-09

**Authors:** Raluca Tutuianu, Ana-Maria Rosca, Daniela Madalina Iacomi, Maya Simionescu, Irina Titorencu

**Affiliations:** Cell and Tissue Engineering Laboratory, Institute of Cellular Biology and Pathology “Nicolae Simionescu”, 050568 Bucharest, Romania; raluca.tutuianu@icbp.ro (R.T.); ana-maria.rosca@icbp.ro (A.-M.R.); madalina.iacomi@icbp.ro (D.M.I.); maya.simionescu@icbp.ro (M.S.)

**Keywords:** exosomes, bone marrow-derived mesenchymal stromal/stem cells, keratinocytes, fibroblasts, angiogenesis, skin organotypic model

## Abstract

Bone marrow-derived mesenchymal stromal cells (MSCs) are major players in regenerative therapies for wound healing via their paracrine activity, mediated partially by exosomes. Our purpose was to test if MSC-derived exosomes could accelerate wound healing by enhancing the biological properties of the main cell types involved in the key phases of this process. Thus, the effects of exosomes on (i) macrophage activation, (ii) angiogenesis, (iii) keratinocytes and dermal fibroblasts proliferation and migration, and (iv) the capacity of myofibroblasts to regulate the turnover of the extracellular matrix were evaluated. The results showed that, although exosomes did not exhibit anti-inflammatory properties, they stimulated angiogenesis. Exposure of keratinocytes and dermal (myo)fibroblasts to exosomes enhanced their proliferation and migratory capacity. Additionally, exosomes prevented the upregulation of gene expression for type I and III collagen, α-smooth muscle actin, and *MMP2* and *14*, and they increased *MMP13* expression during the fibroblast–myofibroblast transition. The regenerative properties of exosomes were validated using a wound healing skin organotypic model, which exhibited full re-epithelialization upon exosomes exposure. In summary, these data indicate that exosomes enhance the biological properties of keratinocytes, fibroblasts, and endothelial cells, thus providing a reliable therapeutic tool for skin regeneration.

## 1. Introduction

Skin wounds represent an unmet therapeutic challenge, with acute forms such as burns, extensive lacerations, as well as chronic lesions often associated with obesity and diabetes [1,2]. The complex process through which a skin lesion is resolved consists of several overlapping phases including inflammation, proliferation, and remodeling, orchestrated by different types of cells such as neutrophils and macrophages, keratinocytes, dermal fibroblasts, and endothelial cells [3].

In recent years, the therapeutic application of bone marrow-derived mesenchymal stromal/stem cells (MSCs) shifted from cellular transplantation to acellular therapy, in light of a novel mechanism of action, based on the favorable effects of factors secreted by these cells. Previous work, including our own, supports the use of MSC conditioned medium (CM) for skin wound healing, on account of its stimulative properties on several key biological processes such as proliferation, migration, and angiogenesis [4,5,6,7,8,9]. Besides growth factors and cytokines, the CM (the secretome) also includes the ubiquitous large family of extracellular vesicles (EVs) comprising exosomes, microvesicles (microparticles), and apoptotic bodies. From this heterogeneous population, exosomes are the smallest vesicles (30–150 nm) of endosomal origin, which act as nanovesicles capable of easily and successfully transferring their cargo (mRNA, microRNA, proteins) to the target cells [10,11]. At the time of writing this paper, around 14 clinical trials currently use exosome administration as a therapeutic approach for various pathologies ranging from respiratory syndromes to cerebrovascular disorders (http://clinicaltrials.gov) (accessed on 14 March 2021). In the case of wound healing, these small nanovesicles are considered to have the capacity to influence key biological processes for skin regeneration [12,13,14,15].

In this study, we questioned whether MSC-derived exosomes (Exo) have a beneficial effect on the four types of cells involved in key phases of the wound healing process, namely macrophages, keratinocytes, dermal fibroblasts, and endothelial cells. We report here that MSC-derived exosomes contribute to wound healing by promoting angiogenesis, stimulating the proliferation and migration of keratinocytes and dermal fibroblasts; preventing the upregulation of gene expression for type I and III collagen, α-smooth muscle actin, and *MMP2* and *14*; and increasing *MMP13* expression during the fibroblast–myofibroblast transition. Furthermore, in a 3D skin organotypic model, the exosomes promoted the complete re-epithelialization of a full-thickness wound.

## 2. Results

### 2.1. Characterization of Exosome-Enriched Suspension Isolated from the Secretome of Human Bone Marrow-Derived Mesenchymal Stromal Cells

**Figure 1:**
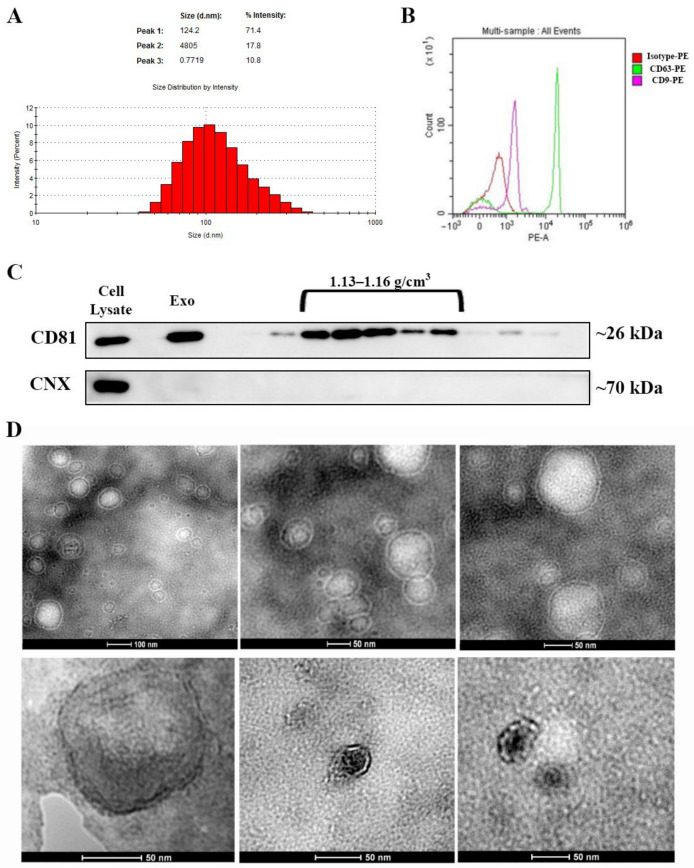
Characterization of exosomes isolated from bone marrow-derived MSC conditioned medium. (**A**) Histogram showing the distribution of the hydrodynamic diameter of the isolated exosomes by dynamic light scattering analysis. (**B**) Expression of exosomal markers CD63 and CD9 as revealed by flow cytometry and (**C**) CD81 as identified via Western blot for the whole pellet (Exo) as well as for the sucrose fractions derived from it. Note the absence of contaminants from the endoplasmic reticulum as indicated by the lack of calnexin (CNX) both in the exosomes (Exo) pellet and the sucrose fractions. (**D**) Transmission electron microscopy images depicting donut-shaped structures up to 100 nm (negative staining with phosphotungstic acid—upper panel and uranyl acetate—lower panel).

Exosomes isolated from serum-free conditioned medium were analyzed for their specific characteristics, in terms of size and protein markers. We found that the isolated vesicle population had a mean hydrodynamic diameter of ~120 nm, as revealed via dynamic light scattering (Figure 1A) and exhibited the presence of two exosomal markers, CD63 and CD9, as evidenced by flow cytometry (Figure 1B). The immunoblot assay divulged the elevated levels of a third exosomal marker-CD81. Furthermore, CD81 was enriched within the whole pellet as well as in the exosomal fractions obtained by sucrose gradient centrifugation, corresponding to a density range of 1.13–1.16 g/cm^3^ (Figure 1C). The pellet, as well as the fractions, were negative for calnexin, suggesting that the Exo were not contaminated with endoplasmic reticulum membranes (Figure 1C). Moreover, the pooled fractions with a density between 1.13 and 1.16 g/cm^3^ examined by transmission electron microscopy displayed the presence of small vesicles, less than 100 nm with a cup-shape morphology specific for exosomes (Figure 1D). Collectively, these data indicated that the MSC-isolated small extracellular vesicles population had the typical characteristics of exosomes, and they were used for further experiments [16,17].

### 2.2. MSC-Derived Exosomes Have No Significant Effect on TNFα Synthesis in LPS-Stimulated Macrophages

**Figure 2:**
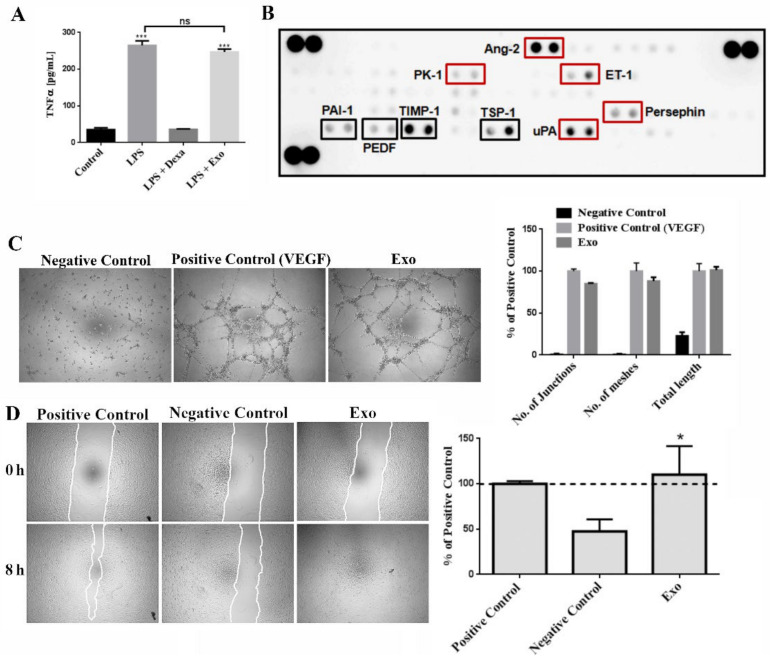
Exosomes have pro-angiogenic effect and do not significantly lower the secretion of TNFα in LPS-stimulated macrophages in vitro. (**A**) Evaluation of the potential anti-inflammatory properties of exosomes as revealed by quantification by ELISA of TNFα in the supernatant of macrophages stimulated with LPS and incubated either with dexamethasone (Dexa) or exosomes (Exo). (**B**) Cytokine array depicting the composition of exosomes regarding the pro- and anti-angiogenic factors, indicated by red and black rectangles, respectively. (**C**) In vitro evaluation of exosomes potential to induce endothelial tube formation on ECM gel substrate. Phase-contrast microscopy images (left, 5× magnification) and graph (right) showing the quantification of the number of junctions, meshes, and total tube length. (**D**) Wound healing assay evaluating the stimulatory effect of exosomes on endothelial cell migration compared to positive (serum) and negative (serum free) controls: phase-contrast microscopy images showing the scratched area at 0 h and 8 h later (left, magnification 5×) and the quantification of covered area as percentage of the positive control (right). Data are means ± SD (*n* = 3), * *p* < 0.05, *** *p* < 0.001, ns—not significant.

To determine whether the Exo have a role in the inflammatory process, we quantified the level of TNFα secreted by LPS-stimulated macrophages (Figure 2A). As expected, the concentration of the inflammatory cytokine in the culture medium harvested from stimulated macrophages reached a significantly higher level (265 ± 12 pg/mL) than in control (unstimulated) cells (35 ± 5 pg/mL). Addition of dexamethasone reversed the LPS effect by lowering the concentration of TNFα to 36 ± 1 pg/mL. The exosomes did not significantly decrease the level of TNFα when compared to control cells (247 ± 8 pg/mL versus 265 ± 12 pg/mL).

### 2.3. MSC-Derived Exosomes Have Pro-Angiogenic Effect

Since angiogenesis is a key step in the wound healing process (proliferative phase), we searched for the presence of cytokines involved in the angiogenic process using a human cytokine array panel. As presented in Figure 2B, the Exo contained both stimulators as well as inhibitors of angiogenesis. The detected stimulators of angiogenesis were angiopoietin-2 (Ang-2), endothelin (ET-1), human endocrine gland derived vascular endothelial growth factor (EG-VEGF/PK1), persephin, and urokinase-type plasminogen activator (uPA). The inhibitors of angiogenesis were thrombospondin-1 (TSP-1), TIMP-1, Serpin-F1 (PEDF), and SERPIN-E1 (PAI-1).

To investigate whether the Exo have indeed a functional angiogenic effect, we used the in vitro tube formation assay of endothelial cells on “ECM Gel Matrix”. Quantification of the number of junctions and meshes, as well as the total tube length, showed that exosomes had a strong pro-angiogenic effect, similar with the VEGF-positive control and in contrast with the negative control (DMEM), where no tube-like structures formation was detected (Figure 2C).

The potential of Exo to influence the migratory properties of endothelial cells was evaluated by scratch test assay. We found that Exo stimulated endothelial cell migration inducing a coverage of the scratched surface area of 110 ± 31%, while the migration in the negative control was 47 ± 13% (Figure 2D). Together, these results were indicative of the pro-angiogenic effect of Exo on human endothelial cells.

### 2.4. MSC-Derived Exosomes Stimulate Proliferation and Migration of Human Keratinocytes and Dermal Fibroblasts

**Figure 3:**
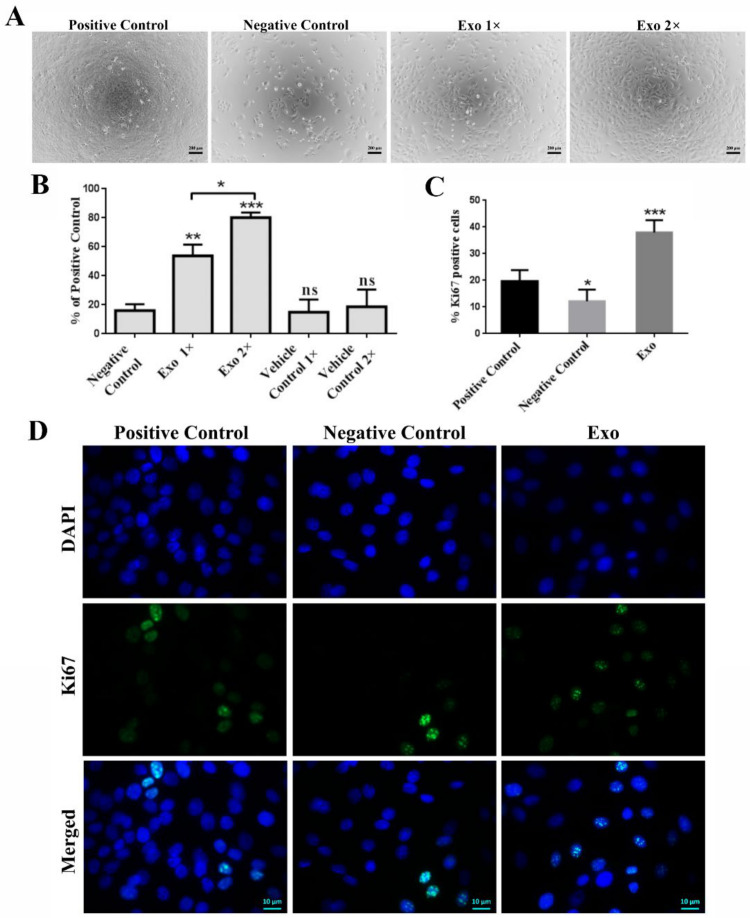
Exosomes enhance the proliferation of HaCaT keratinocytes. (**A**) Phase-contrast microscopy images of the exosomes-treated cells versus controls. (**B**) Assessment of keratinocytes viability by XTT assay performed after 3 days of incubation with two exosomes (Exo) concentrations (1× and 2×). (**C**) Graph showing the quantification of Ki67-positive cells from the total number of cells from each condition resulting from the fluorescence microscopy analysis of keratinocytes grown for 3 days in serum-supplemented medium (positive control), serum-free medium (negative control), and serum-free medium supplemented with exosomes (**D**). Data are means ± SD (*n* ≥ 3), * *p* < 0.05; ** *p* < 0.01, *** *p* < 0.001, ns—not significant.

The effect of Exo on the proliferation of keratinocytes (HaCaT line) and fibroblasts was assessed by incubating the cells for 3 days in the presence of two increasing concentrations of exosomes: 1× (donor to acceptor cells ratio 7:1) and 2× (donor to acceptor cells ratio 14:1). We found that both concentrations of exosomes induced a significant increase in keratinocytes proliferation, namely by 54 ± 8% and 80 ± 4% for 1× and 2×, respectively. These figures were more than double compared to the negative and vehicle controls (~16 ± 4% and 15 ± 9/19 ± 12%, respectively, Figure 3A,B). The detected stimulative effect of exosomes on keratinocytes proliferation was validated by quantifying the nuclear Ki67 staining by immunocytochemistry performed on Exo-treated keratinocytes (Figure 3D). We found that incubation of the cells with Exo induced a higher percentage of Ki67- positive cells (38 ± 7%) than those determined in controls consisting in cells grown in complete medium (positive control, 20 ± 4%) and in serum-free medium (negative control, 12 ± 4%) (Figure 3C,D).

**Figure 4:**
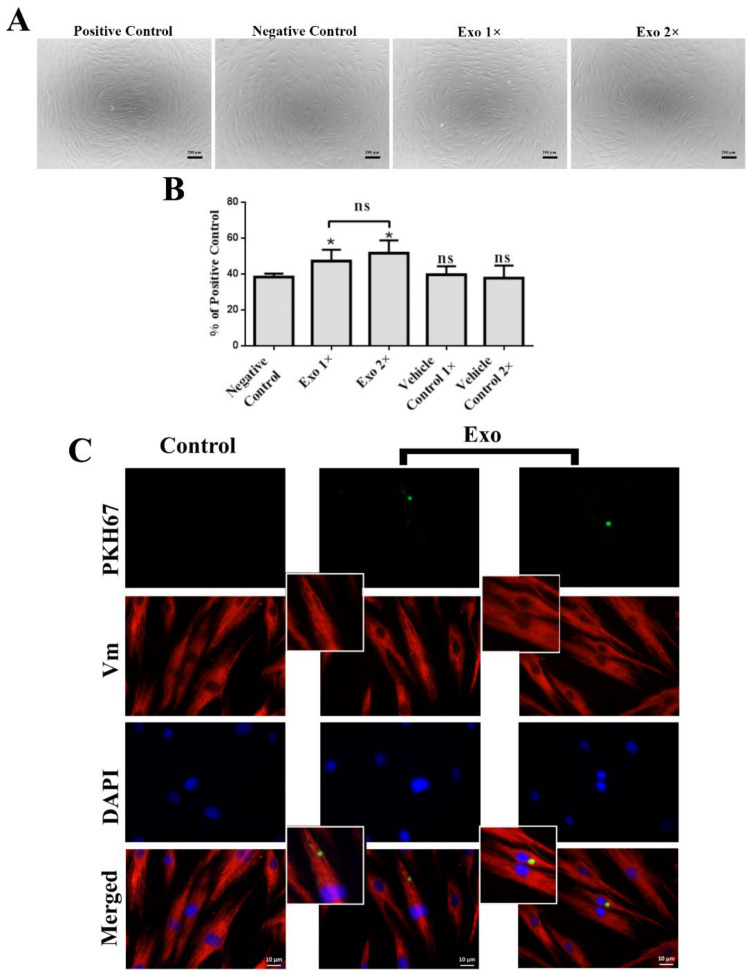
Exosomes enhance the proliferation of human primary dermal fibroblasts. (**A**) Phase-contrast microscopy images of the exosomes-treated fibroblasts versus controls. (**B**) XTT viability assay performed after 3 days of incubation with two exosome (Exo) concentrations (1× and 2×) compared to positive (serum) and negative (serum-free) controls. (**C**) Immunofluorescence images showing the uptake of PKH67-labeled exosomes by dermal fibroblasts. The control consists in fibroblasts incubated with the mock sample stained with PKH67 according to the same procedures as the exosomes pellet. Nuclei were stained with DAPI (blue) and the intermediate filaments with vimentin (red). Data are means ± SD (*n* ≥ 3), * *p* < 0.05, ns—not significant.

Regarding the effect of Exo on the proliferation of human dermal fibroblasts, we found a value of 47 ± 6% in the presence of 1× and 52 ± 7% for 2× concentration of Exo suspension, respectively, which was statistically significant compared to 38 ± 2% for the negative control (Figure 4A,B). However, the impact of both concentrations of Exo used for fibroblasts proliferation was weaker than that obtained for keratinocytes. Therefore, we verified whether this result was a consequence of a poor capacity of fibroblasts to take up exosomes. As shown in Figure 4C, after 4 h of incubation, PKH67-labeled exosomes were readily taken up by the dermal fibroblasts. In a previous study [7], our group obtained similar results for complete MSC-derived conditioned medium, suggesting that this is a characteristic of the whole MSC secretome.

**Figure 5:**
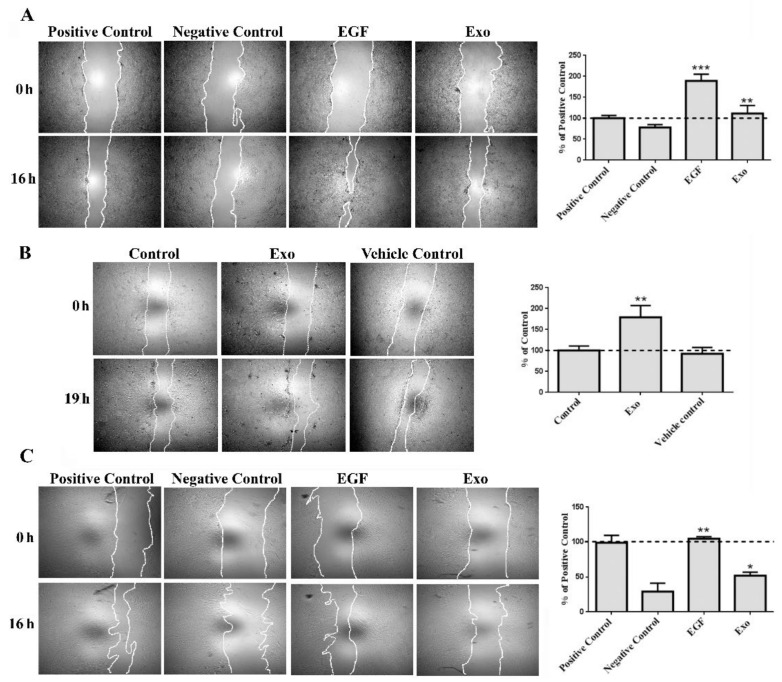
Exosomes stimulate the migration of human HaCaT keratinocytes (**A**), Ntert-1 keratinocytes (**B**), and primary dermal fibroblasts (**C**). Phase-contrast microscopy showing the scratched area at 0 h and 16 h/19 h later (left—magnification 5×), and the quantification of covered area as percentage of the initial scratched area (right), where positive and negative controls are serum and serum-free medium, respectively, for HaCaT and dermal fibroblasts, while control for Ntert-1 keratinocytes is complete medium. Data are means ± SD (*n* ≥ 3), * *p* < 0.05; ** *p* < 0.01, *** *p* < 0.001.

Next, the scratch wound assay was performed in order to evaluate the Exo-stimulative effects on relevant skin cells (1× concentration). For keratinocytes, we employed two immortalized human cell lines, HaCaT and N/TERT-1 keratinocytes, that reportedly are well-known models for in vitro studies of epidermis pathophysiology and pharmaco-toxicology [18,19]. We found that exposure of both cell lines to the Exo induced an increased level of migration. Thus, exosomes enhanced significantly the migration of HaCaT cells to a value of ~111 ± 19% versus 78 ± 7% in the negative control. Since the migration of HaCaT cells in the presence of 10% serum was rather modest, we also employed EGF, a potent mitogen and stimulator of keratinocyte motility. The migration induced by EGF was 189 ± 16%, confirming the validity of the experimental conditions (Figure 5A). In order to verify that this effect was produced by the presence of Exo, we additionally compared the influence of the whole conditioned medium from which the Exo were isolated, as well as the conditioned medium depleted of Exo (Supplementary Figure S1). The results showed that Exo had a higher stimulatory effect in comparison to the extracellular vesicles-depleted medium.

Similarly, in the case of NTERT-1 keratinocytes, Exo were added to the complete culture medium (already containing EGF). The exosomes significantly enhanced the cell motility, which was 179 ± 28% versus 92 ± 15 % for vehicle control (Figure 5B).

The human primary dermal fibroblasts exhibited a migration level of 52 ± 5% upon incubation with exosomes, a value that was significantly higher compared to 29 ± 12% for the negative control. The value obtained for the EGF-stimulated fibroblasts (control) was 105 ± 3% (Figure 5C).

### 2.5. During the Transition of Fibroblasts towards a Myofibroblast Phenotype, MSC-Derived Exosomes Prevent the Upregulation of COL1A1, COL3A1, MMP2, MMP14 as Well as ACTA2 and Increase the Gene Expression of MMP13 

To further investigate the effects of Exo on dermal fibroblasts we employed a culture model relevant for the skin lesion environment, namely a culture medium enriched in TGFβ1. This cytokine is normally found at high levels in the wound bed during the healing process and is a strong inducer of the myofibroblast phenotype [20]. The effect of Exo on the induction of myofibroblasts phenotype was assessed by simultaneous incubation of fibroblasts with TGFβ1 and Exo.

**Figure 6:**
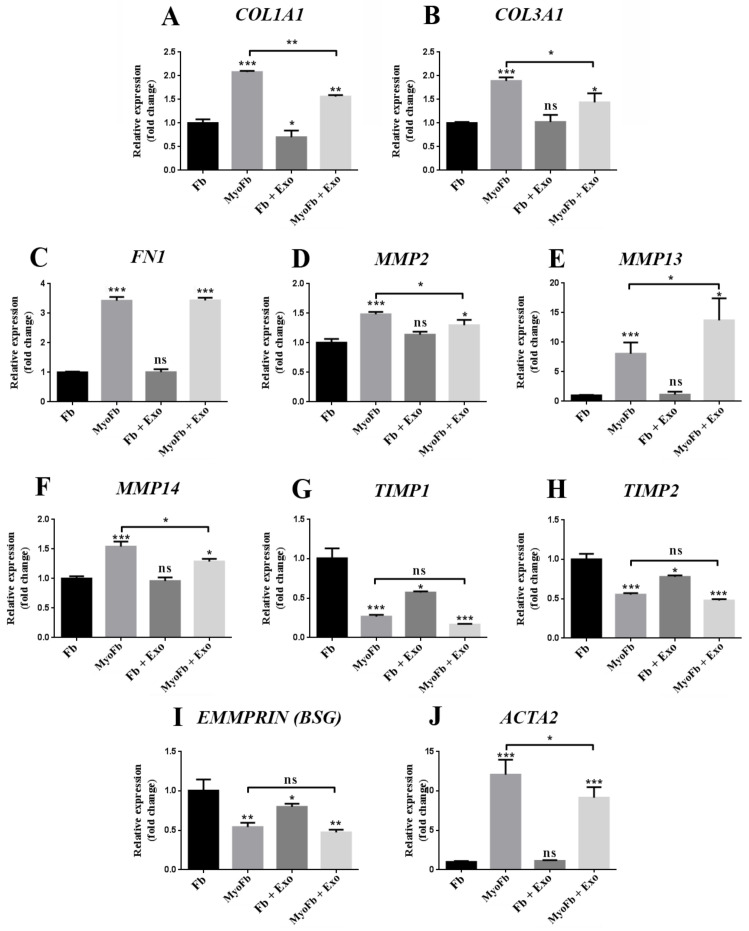
Exosomes modulate the gene expressions of several structural proteins and enzymes involved in matrix remodeling and contractility. The graphs represent the relative mRNA levels of selected genes obtained via qPCR for dermal fibroblasts in normal conditions (control), fibroblasts differentiated towards a myofibroblast phenotype with TGFβ1, fibroblasts incubated with exosomes (Exo), and myofibroblasts exposed to exosomes. *COL1A1*—type I collagen, *COL3A1*—type III collagen, *FN1*—fibronectin, *MMP*—matrix metalloproteinase, *TIMP*—tissue inhibitor of metalloproteinase, *EMMPRIN* (*BSG*)—extracellular matrix metalloproteinase inducer, *ACTA2*—α-smooth muscle actin. Data are means ± SD (*n* = 3), * *p* < 0.05; ** *p* < 0.01, *** *p* < 0.001, ns—not significant.

First, we checked the effect of exosomes on the gene expression of extracellular matrix components such as type I (*COL1A1*) and type III (*COL3A1*) collagen and fibronectin (*FN1*) in fibroblasts and myofibroblasts. In the case of fibroblasts, our results showed that Exo induced a significant decrease (~30%) in the gene expression of *COL1A1*, but it did not affect the expression of *COL3A1* and *FN*1. As expected, the expression of all three genes was upregulated in myofibroblasts where a ~2-fold increase for collagens and 3.4-fold increase for fibronectin were detected. Exo prevented the upregulation of *COL1A1 and COL3A1* (~ 25%) but had no effect on *FN1* expression (Figure 6A,B).

Regarding the gene expression of matrix metalloproteinases (MMPs), exposure of fibroblasts to Exo did not affect the mRNA levels of *MMP2*, *MMP13*, or *MMP14*. Differentiation into myofibroblasts by TGFβ1 treatment led to the upregulation of *MMP2*, *MMP13*, and *MMP14* by 1.5-, 8-, and 1.5-fold, respectively. Exo treatment during myofibroblasts differentiation prevented the increase in mRNA level for *MMP2* and *MMP14* with 13% and 16%, respectively, and upregulated the expression of *MMP13* up to 1.7-fold in comparison to fibroblasts incubated with TGFβ1 alone (Figure 6E,F).

Moreover, the relative mRNA expressions for tissue inhibitors of metalloproteinases (TIMPs) 1 and 2, which regulate the activity of MMPs, were decreased by 1.7- and 1.3-fold in fibroblasts treated with exosomes. The differentiation into myofibroblasts also downregulated the gene expressions of TIMP1 and TIMP2 by 3.7- and 1.8-fold, respectively, and the Exo had no effect on this process (Figure 6G,H).

In fibroblasts, the mRNA level of *EMMPRIN* (*BSG*), which increases the MMPs production in pathological conditions (i.e., tumor invasion in cancer), was decreased by Exo treatment by 1.2-fold. The differentiation of fibroblasts into myofibroblasts also induced a significant decrease in the *EMMPRIN* mRNA level by 2-fold, while the presence of exosomes did not prevent this effect (Figure 6I).

Next, we focused on α-smooth muscle actin (αSMA), which is responsible for the contractile properties of myofibroblasts. As seen in Figure 6, TGFβ1 induced a significant increase in the gene expression of *ACTA2*-alpha smooth muscle actin (12-fold higher than in control fibroblasts). Exo did not modify the gene expression of *ACTA2* in fibroblasts, but they prevented its upregulation during myofibroblast differentiation by ~25% (Figure 6J).

### 2.6. MSC-Derived Exosomes Increase the Protein Expression of Type I Collagen during the Transition of Fibroblasts into Myofibroblasts and Increase the Expression of Decorin and Fibronectin in Fibroblasts

**Figure 7:**
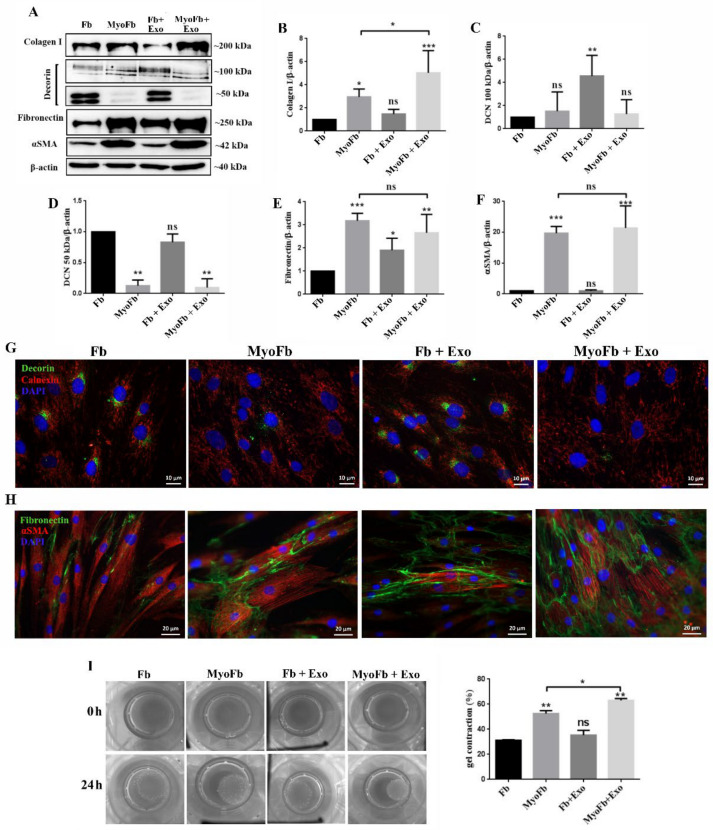
Exosomes stimulate the expression of proteins involved in matrix remodeling and contraction in fibroblasts differentiated towards myofibroblasts. (**A**) Representative Western blots images and quantification relative to β-actin for type I collagen (**B**), decorin (**C**,**D**), fibronectin (**E**), and αSMA (**F**). (**G**) Immunocytochemistry image indicating the changes of decorin in fibroblasts treated with TGFβ1, exosomes, TGFβ1 and exosomes, and its distribution in relation to the endoplasmic reticulum evidenced by calnexin. (**H**) Fluorescence microscopy image showing the organization of αSMA in stress fibers in TGFβ1-treated samples and the extracellular organization of fibronectin. (**I**) Gel contraction assay demonstrating the contractile activity of fibroblasts as such (control), differentiated (TGFβ1), incubated with exosomes (Exo), and simultaneously stimulated with TGFβ1 and exosomes (TGFβ1 + Exo). The results are given as the percentage of the initial area of the gel. Data are means ± SD (*n* = 3), * *p* < 0.05; ** *p* < 0.01, *** *p* < 0.001, ns—not significant.

We assessed the Exo effect on the protein expression of several extracellular matrix components and the contractile protein αSMA (alpha smooth muscle actin) in human dermal fibroblasts, and those subjected to myofibroblasts differentiation by TGFβ1 treatment. As expected, treatment of the cells with TGFβ1 induced a ~2.6-fold increase above the control for type I collagen (collagen I), a feature that was not affected by Exo exposure. However, the latter induced a significant increase in differentiated myofibroblasts (TGFβ1 + Exo), for which the protein level was ~1.7-fold higher versus myofibroblasts (Figure 7A,B).

Next, the expression of decorin, a collagen-associated extracellular matrix proteoglycan, was assessed, for both the glycosylated (100 kD) and unglycosylated (50 kD) forms. The results showed that Exo induced a significant 4.5-fold increase in the expression of the glycosylated decorin in fibroblasts; no differences were observed in myofibroblasts, in the absence or presence of exosomes (Figure 7C). In contrast, the expression of the intracellular non-glycosylated form was strongly downregulated in myofibroblasts (0.12-fold), and the exosomes did not prevent this process, as revealed both by Western blot (Figure 7D) and immunocytochemistry (Figure 7G).

Regarding fibronectin, all treatments (TGFβ1, Exo, or their combination) induced an increase at the protein level of ~3-, ~1.9-, and 2.7-fold, respectively, as showed by Western blot (Figure 7E) and immunocytochemistry (Figure 7H). We found that, in this case, exosomes had no impact on the fibronectin expression in myofibroblasts.

Concerning αSMA, as expected, the differentiation of fibroblasts by TGFβ1 treatment induced a significant increase in the protein expression (~19-fold). The exosomes did not change the expression of this protein, nor did they prevent its upregulation during differentiation into myofibroblasts (Figure 7A,F). This tendency was validated by the flow cytometry analysis (Supplementary Figure S2). Moreover, the organization of αSMA in stress fibers, as revealed by immunocytochemical staining, did not show differences between the fibroblasts stimulated with TGFβ1 alone and those exposed to both exosomes and the growth factor (Figure 7H).

Furthermore, in order to functionally assess the effect of exosomes on fibroblasts contractility, we performed the collagen gel contraction assay. The results indicated that the ability of fibroblasts to contract the matrix was similar irrespective of the exposure to Exo (35 ± 4% for exosome-treated fibroblasts and 31 ± 0.5% for fibroblasts). In contrast, in the case of myofibroblasts, these nanovesicles promoted contractility, manifested by significantly higher reduction in the gel surface area: 62 ± 1% compared to 53 ± 2% for myofibroblasts untreated with exosomes.

Since the αSMA level was similar in myofibroblasts exposed or not to Exo, we checked the expression of lisyl oxidase (LOX), an enzyme responsible for collagen cross-linking. The results showed a significant increase in LOX protein expression: 17.4-fold and 8.4-fold for the immature (pro-LOX) and the mature form of LOX in Exo treated myofibroblasts versus myofibroblasts (Supplementary Figure S3), thus suggesting a cause for the increase in cell contractility for the combined condition.

### 2.7. MSC-Derived Exosomes Stimulate Wound Re-Epithelialization

**Figure 8:**
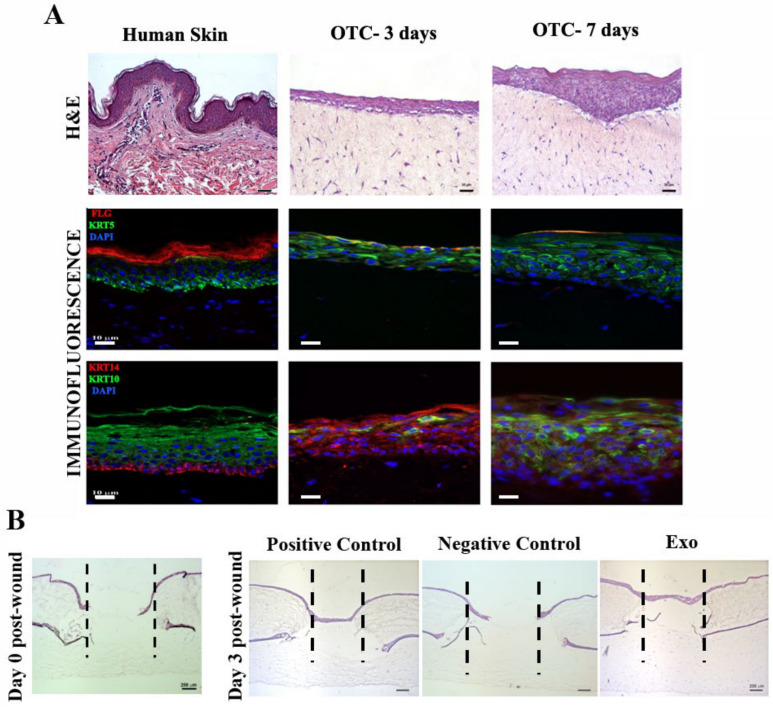
Exosomes support wound healing on a human skin organotypic model. (**A**) Histological (hematoxylin and eosin staining) and immunofluorescence images showing the skin-like structure of the organotypic culture versus human adult skin tissue (left). Note the expression of keratinocytes markers fillagrin (FLG) and keratins (KRT) 5, 10, and 14, similar to the skin control. (**B**) Hematoxylin and eosin staining showing the healing of the skin organotypic cultures: left, image taken immediately after performing the punch wound (day 0 post-wound); right, after 3 days for the samples incubated with complete medium (positive control), basal medium (negative control), and exosomes (Exo).

To validate the positive results obtained upon exposure of Exo to cultured keratinocytes and fibroblasts, a skin organotypic equivalent, consisting in a coculture of human keratinocytes and dermal fibroblasts within a three-dimensional collagen matrix, was employed. Our construct exhibited a suitable thickness of the epidermal layer and expressed the markers for basal keratinocytes (keratin 5/14) as well as early (keratin 10) and late (fillagrin) differentiation during the maturation process, similar to the native skin (Figure 8A). After performing full-thickness wounds, the organotypic cultures were incubated either with complete culture medium, basal medium (without serum and growth factor supplementation), or basal medium containing exosomes. We found that three days after injury for both Exo and positive control samples, the wounded areas were covered with a continuous layer of keratinocytes, as revealed by the hematoxylin and eosin histological staining, unlike the still incomplete epidermis for the negative control (Figure 8B). These results were in good agreement and validated the data obtained in 2D cultures, thus supporting that MSC-derived exosomes have a significant regenerative effect on keratinocytes.

## 3. Discussion

Cell-free regenerative medicine has recently gained a fresh impetus. Among others, the focus centers on the mesenchymal stromal cells-derived exosomes as the main effectors of the paracrine activity exerted by these cells. In a previous study, we have reported MSC secretome to be relevant for skin wound healing by promoting colonization of collagen scaffolds [7]. Since little is known about the particular secretome components that influence the process of wound healing, we set up a series of experiments to investigate the specific role of exosomes in this process, in particular during the main phases of the skin healing process: inflammation, proliferation, and maturation.

The initial inflammation is a key phase in the wound healing process, which partially triggers the angiogenic response by producing high levels of VEGF [21,22]. Macrophages have a controversial role in the wound healing process: they can influence the outcome either towards a minimum scar tissue or to an impaired healing wound, as a function of the balance between the occurrence of inflammatory (M1) and reparatory (M2) macrophage phenotype [23,24]. In vivo administration of MSC isolated from the dermal and adipose tissues on a mouse skin wound model has been associated with a reduction in inflammation by switching the M1 macrophages towards the reparative M2 phenotype [25]. In addition, in a recent paper investigating the role of bone marrow MSC-derived exosomes on the tendon-bone healing, it was reported that, besides their angiogenic stimulative action, the exosomes had an anti-inflammatory effect by inhibiting the secretion of IL-1β, TNF-α, IL-6, and IL-8 by M1 macrophages [26]. In contrast to these data, we did not find a significant reduction in the level of the inflammatory cytokine TNFα in activated macrophages treated with exosomes. This result is in agreement with a previous report, which highlighted that conditioned medium secreted by 3D spheroid culture, but not 2D adherent bone marrow-derived MSCs, had anti-inflammatory effects in the same experimental setting as ours [27]. The data showed the important role of the culture system in conferring certain therapeutic features to MSC-derived products. Nevertheless, Zhang and collaborators showed that the initial inflammation is beneficial for the resolution of the lesion [28]; thus, using MSC-derived exosomes as treatment for skin injuries can positively impact the progression of the normal healing process.

Angiogenesis represents a critical step occurring in the proliferative phase of the wound healing process. When searching for the effect of exosomes on endothelial cells (ECs), we found that they stimulate both the migration and the tube-like formation. These data confirmed and extended previous reports obtained with exosomes derived from bone marrow MSCs as well as umbilical cord MSCs [29,30]. Interestingly, we found that exosomes contain both pro- (Ang-2, ET-1, EG-VEGF/PK1, persephin, uPA) and anti-angiogenic factors (TSP-1, TIMP-1, PEDF, PAI-1). This suggests that these types of extracellular vesicles can play a role in sustaining the angiogenic balance between the induction and prevention of this process. It is worth mentioning that some of these factors, such as TSP-1 and Ang-2, implicated in the maturation and stabilization of blood vessels, are downregulated in diabetic wounds [31]. This indicates that for the patients with this pathology, exosomes delivery to the wound site can be highly beneficial.

Regarding the proliferation and migration of keratinocytes and fibroblasts, we found that exosomes secreted by bone marrow-derived MSCs significantly increase both processes in these cells. These results corroborate well and extend previous reports on exosomes obtained from other sources such as adipose-tissue-derived MSCs [32,33,34] as well as bone marrow-derived MSCs [35]. It is known that in chronic wounds the cells residing in the periphery of the lesion have low proliferative and migratory capacity [36]. Therefore, in addition to stimulating angiogenesis, exosomes can also be valuable to enhance the expansion and migratory properties of skin cells in impaired wound healing.

The maturation phase, which partially overlaps with the proliferation stage, is mainly orchestrated by fibroblasts and myofibroblasts, the providers of matrix deposition, remodeling, and wound contraction [37]. Among the cytokines identified within the wound bed during these processes, TGFβ1, the prototype member of the TGFβ family, is considered to have a major role in the regenerative outcome [38,39,40], being involved in fibroblast–myofibroblast transition. In our experiments, we searched for the influence of MSC-derived exosomes on the capacity of fibroblasts/differentiating myofibroblasts to control the turnover of the main extracellular matrix proteins. Since the exaggerated responses to fibrogenic cytokines such as TGFβ1 might induce an aberrant fibroblast phenotype involved in the formation of scars [41], we investigated whether exosomes contributed to this undesired side effect.

Thus, in the case of fibroblasts, the exosomes had little or no effect on the gene expressions of *COL1A1* and *COL3A1*, while they partially downregulated the transcription of these genes during myofibroblast differentiation. Regarding the protein expression of type I collagen in differentiated myofibroblasts, the presence of exosomes increased significantly this protein in comparison to untreated myofibroblasts. In pathological conditions, such as hypertrophic scars, collagen production is exacerbated. In our hands, MSC-derived exosomes did not induce a fibrotic phenotype in fibroblasts.

Fibronectin is implicated in all stages of wound healing [42], but its excessive deposition could contribute to scar formation [43]. In our experiments, the gene expression of fibronectin was not modified by exosomes treatment in any condition. However, the fibronectin protein expression was significantly increased in fibroblasts incubated with exosomes, suggesting the induction of a pro-healing phenotype. Concurrently, the presence of exosomes did not impact the protein expression of fibronectin during myofibroblasts differentiation (which correlates well with the gene expression), indicating that this approach does not promote an aberrant phenotype of these cells. Similarly, the same pattern was observed for the glycosylated decorin, whose protein expression was increased in fibroblasts by exosomes treatment. Decorin is known to prevent tissue fibrosis and promote tissue regeneration [44], which is also involved in ECM assembly [45]. Therefore, our data suggest that MSC-derived exosomes, by their effects on fibroblasts, support a normal healing process.

Specific proteases, such as MMPs, mediate collagen remodeling during the wound healing process [46]. Here we showed that exosomes treatment was not associated with a modification in the gene expressions of *MMP2*, *13*, and *14* in fibroblasts, but it did alter the tendency of upregulation induced by TGFβ1 during myofibroblast transition. Thus, in the case of *MMP2* (gelatinase A, found at high levels in the fluid of human chronic wounds [47]) and *MMP14* (membrane type 1 MMP, a collagenolytic enzyme responsible for collagen remodeling), exosomes partially prevented the upregulation of their mRNA level, while for *MMP13* (an interstitial collagenase known to enhance the motility and contractility force generated in fibroblasts [48]) the upregulation was promoted compared to untreated myofibroblasts. Interestingly, for *TIMP1* and *2*, which inhibit these three MMPs, their gene expression was downregulated in fibroblasts by the Exo, but was not modified during myofibroblast differentiation. These results suggest that the effect of MSC-derived exosomes on dermal fibroblasts, even in a TGFβ1-rich environment, does not shift the balance towards a pro-fibrotic phenotype but rather to a pro-healing phenotype.

Regarding αSMA, considered a marker for myofibroblast differentiation, exosome treatment did not have any effect, at both gene and protein levels, in dermal fibroblasts, while partially preventing the upregulation of the mRNA level during myofibroblast differentiation. However, this tendency was not observed at the protein level, suggesting that Exo treatment does not impede wound contraction and closure and does not promote a pro-fibrotic effect even in the presence of TGFβ1. Moreover, we observed an increase in cell contractility in the case of Exo-stimulated myofibroblasts compared to the untreated myofibroblasts, which may be due to an upregulation in the expression of LOX, an enzyme involved in the cross-linking of collagen fibers and wound contraction [49].

Several papers reported the beneficial action of MSC-derived exosomes isolated from various sources, including bone marrow, on animal skin full-thickness or burn wound models [30,50,51,52]. However, these in vivo studies have been performed on rodents, either mouse or rat, which, due to anatomical and physiological differences, display little concordance with the results observed in humans [53]. Therefore, we chose to validate our results obtained in 2D culture on a more clinically relevant 3D model: a human skin organotypic culture. This type of skin model including a reconstructed human epidermis is already used as a standard for skin irritation testing according to ISO 10993-23:2021-Part 23. Our results showed that MSC-derived exosomes stimulated the re-epithelialization process on the wounded human skin organotypic model, similar to the complete medium supplemented with the appropriate growth factors, reinforcing the beneficial use of these small extracellular vesicles for tissue regeneration.

In conclusion, exosomes secreted by human bone marrow-derived MSCs stimulate key biological processes in the main cell types involved in the major stages of the skin wound healing process. Exosome treatment (i) did not impede the release of TNFα in the inflammatory stage of wound healing, which is an important first step; (ii) it stimulated the proliferation and migration of keratinocytes and dermal fibroblasts, as well as (iii) enhanced angiogenesis, all important features of the proliferative phase; and (iv) it modulated the expressions of crucial proteins associated with the remodeling phase (extracellular matrix components, MMPs and TIMPs). Importantly, exosomes ensured the complete re-epithelialization of a full-thickness lesion produced on a human three-dimensional skin organotypic model. Together, these data provide novel insights into the regenerative properties of small extracellular vesicles, indicating that MSC-derived exosome-based therapy for the treatment of skin wounds can be valuable when applied in any phase of the healing process, starting with the inflammatory stage.

## 4. Materials and Methods

### 4.1. Cell Culture

Human MSC and dermal fibroblasts were isolated after informed consent with the approval of the Institutional Ethical Committee (180/27 September 2018), in accordance with the most recent version of the Helsinki declaration of World Medical Association (Ethical Principles for Medical Research Involving Human Subjects, October 2008). The cells were characterized as previously described [7]. Both cell types were plated at 10^4^ cell/cm^2^ and grown in DMEM 1 g/L glucose supplemented with 1% non-essential amino acids (Sigma Aldrich, St. Louis, MO, USA), 10% fetal bovine serum (FBS), 1% penicillin, streptomycin and neomycin, at 37 °C and 5% CO_2_.

Human keratinocyte cell line HaCaT (CLS GmbH, Germany) and human endothelial cell line EA.hy926 (ATCC CRL-2922) were employed according to the manufacturer’s instructions. Briefly, the cells were cultivated in DMEM (Sigma Aldrich, St. Louis, MO, USA) with 1% non-essential amino acids (Sigma Aldrich, St. Louis, MO, USA) and 10% FBS, at 37 °C, 5% CO_2_.

The human keratinocyte cell line Ntert-1 Ker was a kind gift from Ellen van de Bogaard (Radboud University Medical Center) with the approval of prof. J. G. Rheinwald and was used as previously described [54]. Briefly, the cells were cultured in K-SFM (Gibco, Waltham, MA, USA) until reaching 50% confluence, after which the medium was changed to a mixture of K-SFM and DF-K at 1:1 (vol.vol), with DF-K being DMEM:F12 (Gibco, Waltham, MA, USA) at 1:1, supplemented with 25 µg/mL BPE (bovine pituitary extract), 2mM L-glutamine, 0.2 ng/mL EGF, and 300 µM CaCl_2_, all from Gibco, in order to obtain a confluent monolayer.

For qPCR, Western blot, and collagen gel contraction assays, dermal fibroblasts were seeded on 24-well plates at 10^4^ cells/cm^2^ in complete medium, and after 24 h the cells were washed with PBS and starved in DMEM containing 0.1% exosome-depleted FBS. After another 24 h, the cells were incubated in DMEM supplemented with 10 ng/mL TGFβ1 (Gibco, Waltham, MA, USA) in order to induce the myofibroblasts phenotype in the presence or absence of exosomes. Fibroblasts incubated in DMEM were used as control. All samples contained 0.5% exosome-depleted FBS in order to ensure the growth factors necessary for cell survival.

### 4.2. Exosomes Isolation and Characterization

Pre-confluent MSCs at passages 5–7 were incubated in serum-free DMEM for 24 h. Then, the conditioned medium (secretome) was collected and processed as previously described [55]. Briefly, the secretome was centrifuged as follows: 2 × 300 *g*, 2 × 500 *g*, 10 min each, at 4 °C, followed by a 0.22 µm filtration. The resulted pre-cleared medium was ultracentrifuged at 100,000× *g* (average) for 18 h, at 4 °C, using a Beckman Coulter ultracentrifuge and a Ti50.2 rotor. The pellet was washed for 4h in PBS, then resuspended in PBS and stored at −80 °C. The MSCs were counted for each harvest, and the number was used to establish the amount of exosome suspension to be added for the functional assays, so that the donor-to-acceptor cell ratio will be 7:1.

The size of the isolated MSC-derived exosomes (Exo) resuspended in PBS was determined using a Malvern Nanosizer, with the following parameters: viscosity—0.8882cP and refractive index—1.335.

Exosomes were characterized for the presence of CD9, CD81, and CD63. These exosomal markers were evidenced by flow cytometry and Western blot. Human CD63 Isolation/Detection kit (Invitrogen, Waltham, MA, USA) was used according to the manufacturer’s instructions to isolate the vesicles on Dynabeads, which were further stained with the following antibodies: CD63 Monoclonal Antibody (H5C6), PE, (eBioscience, San Diego, CA, USA) and CD9 Monoclonal Antibody (SN4 C3-3A2), PE, (eBioscience). The samples were analyzed using a Beckman Coulter 3 laser CytoFLEX flow cytometer, and the data were analyzed with CytExpert software v2.1 (Beckman Coulter, Indianapolis, IN, USA).

To determine the lack of contaminants and to verify the density of the isolated vesicles, we employed Western blot assay. The pellet was either directly lysed in Laemmli Sample Buffer without β-mercaptoethanol or underwent a sucrose gradient (concentration range 0.4-2 M) ultracentrifugation (200,000× *g*, SW55Ti rotor), as previously described [56]. The sucrose fractions were collected individually and lysed with the same buffer. The cell lysate (MSC from which conditioned medium was harvested), the whole exosome pellet, as well as the sucrose fractions were analyzed as described in the Western blot section.

For transmission electron microscopy, the exosomes were fixed in 2% PFA and deposited on formvar carbon-coated copper grids for 20 min, followed by negative staining with 1% uranyl acetate (2 min) or 2% phosphotungstic acid (pH~7, 30 s). After drying on filter paper, the grids were examined using a FEI TECNAI F20 electron microscope.

### 4.3. Effect of Exosomes on Inflammatory Macrophages

Human monocytic cells U937 were differentiated towards macrophages following a previously published protocol [27]. Briefly, 5 × 10^5^ cells/mL were cultured in RPMI-1640 with 10% heat-inactivated FBS and 100 ng/mL PMA (phorbol-12 myristate 13-acetate, Sigma Aldrich, St. Louis, MO, USA) for 48 h, and then the culture medium was changed. After 24 h, the cells were gently scraped and treated with 100 ng/mL LPS (Sigma Aldrich, St. Louis, MO, USA) for 10 min. The cells (un-stimulated and stimulated) were then seeded in 24-well plates and incubated with exosomes or vehicle (control) for 18 h, after which the medium was collected and the TNFα concentration was quantified using a human TNFα Quantikine ELISA kit (R&D Systems, Minneapolis, MN, USA).

### 4.4. Tube Formation Assay

Endothelial cells (EAhy926 line) were seeded in 96-well plates on 50 μL of ECM Gel Matrix (Sigma Aldrich, St. Louis, MO, USA) at a density of 45,000 cells/cm^2^ in either basal medium without serum (negative control), basal medium supplemented with 50 ng/mL VEGF (positive control), or with Exo. Images were taken after 16 h using a Zeiss Axiovert microscope (5× magnification) and processed with ImageJ software (NIH, Bethesda, MD, USA).

### 4.5. Viability Assessment

Cells were seeded in complete medium on 96-well plates, and after 24 h, the medium was changed to serum free DMEM with 1% non-essential amino acids (negative control), DMEM with 1% non-essential amino acids and 10% serum (positive control), and DMEM with 1% non-essential amino acids and exosomes (Exo). The cells were incubated for 3 days in the presence of two increasing concentrations of Exo: 1× (donor-to-acceptor cell ratio 7:1) and 2× (donor-to-acceptor cell ratio 14:1). Cell proliferation was evaluated using the XTT assay viability/proliferation kit (Thermo Scientific, Waltham, MA, USA), according to the manufacturer’s instructions, and the results were reported as percentage of the positive control.

### 4.6. Cellular Uptake of Exosomes

The exosomes were stained with PKH67 Green Fluorescent Cell Linker Kit (Sigma Aldrich, St. Louis, MO, USA) following the manufacturer’s instructions. Briefly, after isolation via ultracentrifugation (as described above), the PBS resuspended exosomes and serum-free media (negative control) were incubated with 1 mL Diluent C with 6 μL PKH67 for 5 min. The excess dye was bound with 2 mL 10% BSA and diluted with serum-free medium, after which a 0.971M sucrose solution was added slowly into the tube. Following ultracentrifugation at 190,000× *g* (2 h, 4 °C), the pellets were resuspended in PBS, transferred to 10 kDa MWCO filter columns (Amicon, Merck Millipore, Burlington, MA, USA) and spun at 3000 g, 30 min, 4 °C to a final volume of 1 mL. Fibroblasts grown on glass coverslips for 24 h were incubated with either PKH67 label exosomes or negative control for 18 h and then processed via immunocytochemistry for vimentin staining.

### 4.7. Scratch Wound Assay

Keratinocytes (Ntert-1 Ker and HaCaT), dermal fibroblasts, and endothelial cells (EAhy926) were grown in 96-well plates and, upon reaching confluence, a lesion was performed on the monolayer using a 200 μL pipette tip. In order to distinguish between proliferation and migration, the cells were serum-starved for 24 h before the lesion was performed. After washing the debris with PBS, the cells were incubated as described in Section 4.5: serum-free DMEM (negative control), DMEM with 10% serum (positive control), and DMEM with exosomes. In the case of HaCaT and dermal fibroblasts the effect of 5 ng/mL EGF (epidermal growth factor) was also tested; for Ntert-1 Ker the exosomes were added in their corresponding complete serum-free medium (the mixture of KSFM and DF-K), since these cells did not survive for the length of the experiment in medium without growth factors. The cells were photographed immediately after the addition of the media—0 h and at 16 h for HaCaT and dermal fibroblasts, and 19 h for Ntert-1 Ker. The migration of the cells was quantified by measuring the scratched area covered by the cells using ImageJ software (NIH, Bethesda, MD, USA). The data were standardized considering the positive control as 100%.

### 4.8. Quantitative Real-Time PCR

Total RNA was extracted from fibroblasts using the TRIzol reagent (Thermo Fisher Scientific), and cDNA was synthesized starting from 1 μg of total RNA employing SENSIFAST cDNA Synthesis Kit (Bioline, Cincinnati, OH, USA). Real-time PCR was performed using The SensiFAST™ SYBR Hi-ROX Kit (Bioline, Cincinnati, OH, USA) optimized amplification conditions on the Applied Biosystems ViiA7 Real-Time PCR system (Applied Biosystems, Foster City, CA, USA). The experiments were performed three times in triplicate for each gene. The primer sequences are given in the Supplementary Materials (Table S1). The analysis was done using the comparative CT method, and GAPDH was employed for internal normalization.

### 4.9. Western Blot

Cells were lysed using Laemmli Sample Buffer (Sigma Aldrich, St. Louis, MO, USA) containing a protease inhibitors cocktail (Sigma Aldrich, St. Louis, MO, USA), and 10 µg/mL total protein was separated on 10% polyacrylamide gel via SDS-PAGE, followed by transfer on methanol-activated PVDF membrane (Merck Millipore, Burlington, MA, USA). The membranes were blocked in 5% non-fat powdered milk for 1 h at room temperature, after which they were incubated with primary antibodies to type I collagen (PA5-29569, ThermoFisher Scientific), fibronectin (PA5-29578, ThermoFisher Scientific), αSMA (14-9760-82, Invitrogen, Waltham, MA, USA), and β-actin (A5441, Sigma Aldrich, St. Louis, MO, USA), overnight, at 4 °C. The next day, after 3 washes with PBST, the membranes were incubated with the corresponding secondary antibodies: goat α-mouse HRP or goat α-rabbit HRP (Thermo Scientific) for 1 h at room temperature. The membranes were developed with Immobilon Forte HRP Substrate (Merck Millipore, Burlington, MA, USA) using a Luminescent Image analyzer LAS-3000 (FUJIFILM, Japan). The relative protein expression was quantified via densitometric analysis with the Scion Image software.

For the exosomal markers analysis, the samples were subject to non-reducing electrophoresis, and the following primary antibodies were used: CD81 (MA5-13548, ThermoFisher) and calnexin (MA3-027, Invitrogen, Waltham, MA, USA), following the same procedure as above.

### 4.10. Collagen Gel Contraction Assay

Fibroblasts, pre-treated as described above (control, TGFβ1, exosome, TGFβ1+exosomes), were embedded in collagen gel (rat tail, Merck Millipore, Burlington, MA, USA) at a final concentration of 1mg/mL following a previously published protocol [57]. Briefly, 500 μL of collagen gel containing 5 × 10^4^ fibroblasts was cast in triplicate for each condition in 24-well plates. After solidification, the gels were incubated in DMEM with 0.5% exosome-depleted FBS and dissociated from the wells using a pipet tip so that the structures were floating in the liquid. The gels were photographed at 0 h and 24 h, in order to determine the surface area, using ImageJ (NIH, Bethesda, MD, USA). The contraction was reported using the following formula:

(A0 − Af)/A0 × 100,

where A0 and Af represent the initial and final surface area of the gel.

### 4.11. Human Skin Organotypic Culture

Human skin organotypic cultures/equivalents were established in previously published protocols [58,59]. Briefly, the dermal equivalents were obtained by mixing 1 volume of FBS containing 3.75 × 10^5^/mL dermal fibroblasts with 1 volume 10× Hank’s buffered saline (Sigma Aldrich, St. Louis, MO, USA) and 8 volumes of bovine type I collagen (Thermo Scientific), neutralized with 2M NaOH. The gel was cast on 24-well polycarbonate inserts (Merck Millipore, Burlington, MA, USA), and DMEM with 10% FBS was added in both compartments for 24 h. The next day, HaCaT (10^5^ cells/insert) cells were seeded on top of the collagen layer. After 4 days, the construct was raised to the air–liquid interface, and the medium was supplemented with 2 ng/mL TGFα, 100 ng/mL GM-CSF, and ascorbic acid to a final concentration of 50 mg/L. After 7 days, using a 2 mm biopsy punch, a full-thickness lesion was made on the constructs that were moved on top of another collagen layer containing fibroblasts. The skin equivalents were fixed in 4% PFA at day 0 and day 3 post wound, and processed for cryosectioning and hematoxylin and eosin staining.

### 4.12. Immunofluorescence

Cells (fibroblasts/HaCaT) cultured on glass coverslips were fixed and permeabilized in 4% PFA with 0.1% Triton X and blocked with 1% BSA, before staining with the primary antibodies for type I Collagen (PA5-29569, ThermoFisher Scientific), fibronectin (PA5-29578, ThermoFisher Scientific), αSMA (14-9760-82, Invitrogen, Waltham, MA, USA), Ki67 (PA5-19462, Invitrogen, Waltham, MA, USA), and vimentin (V2258, Sigma Aldrich, St. Louis, MO, USA). After washing, the cells were incubated with secondary antibodies conjugated with Alexa 488 or Alexa 568 (Thermo Scientific). The coverslips were mounted with Fluoroshield with DAPI (Thermo Scientific) and visualized using a Zeiss Observer D1 microscope.

For the skin organotypic cultures, 5 μm frozen sections fixed in ice-cold methanol were blocked with 1% gelatin and then stained with antibodies for keratin 5 (MA5-16372), keratin 10 (MA1-06312), keratin 14 (MA5-11599), and fillagrin (MA5-13440), all from Thermo Scientific.

### 4.13. Statistical Analysis

Data are presented as mean ± standard deviation (SD) from at least three independent experiments, measured as triplicates, and statistical analysis was performed with the GraphPad Prism 6 software (GraphPad, San Diego, CA, USA). Comparison of multiple groups was done by ANOVA with Bonferroni’s multiple comparison test (*, *p* < 0.05, **, *p* < 0.01, ***, *p* < 0.001, ns, not significant).

## Data Availability

Data available on request from the authors.

## Supplementary Materials

The Supplementary Materials are available online at https://www.mdpi.com/article/10.3390/ijms22126239/s1.
