# Supplementary material for: Human Mesenchymal Stromal Cell-Derived Exosomes Promote In Vitro Wound Healing by Modulating the Biological Properties of Skin Keratinocytes and Fibroblasts and Stimulating Angiogenesis
Source: Int J Mol Sci. 2021 Jun 9;22(12):6239. doi: 10.3390/ijms22126239 (PMC8228793; doi:10.3390/ijms22126239)
Supplement: Supplementary file 1 [file ijms-22-06239-s001.zip › ijms-1237138-supplementary.pdf]

Supplemental material

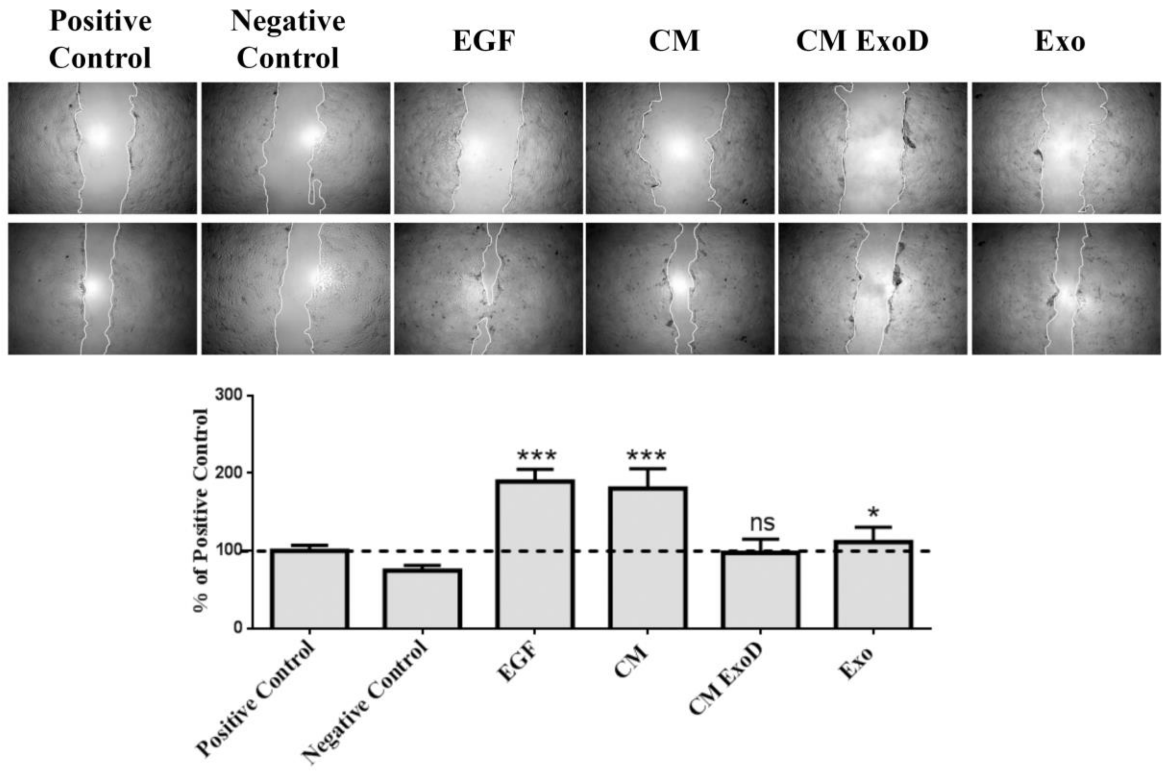

Figure S1. Migratory capacity of HaCaT keratinocytes in serum supplemented culture medium (positive control), serum free culture medium (negative control), serum free culture medium supplemented with epidermal growth factor (EGF), conditioned medium (CM), conditioned medium depleted of exosomes (CM ExoD) and serum free medium supplemented with exosomes (Exo): upper panel- phase contrast images, lower panel- quantification reported as percentage of positive control. Notice that conditioned medium and EGF supplemented conditions presented similar stimulatory activity, and Exo- although lower than the conditioned medium from which they were isolated, it was higher than the depleted condition.

Dermal fibroblasts grown as described in section 4.1. were detached with trypsin and fixed with 4% PFA for 10 minutes, followed by permeabilization with ice-cold methanol for another 10 minutes. The samples were washed with PBS containing 2% FBS and 2mM EDTA and blocked with 10% FBS for 15 minutes. The cells were incubated with the primary antibody- αSMA (Invitrogen) for 30 minutes. Following another wash, the second antibody – Alexa 568 conjugated goat anti-mouse (Invitrogen) was added for another 30 minutes. After a final wash, the samples containing 10^5 cells were run on a Beckman Coulter 3 laser Gallios cytometer and the data were analyzed with the Summit software v4.3 (Cytomation, Inc).

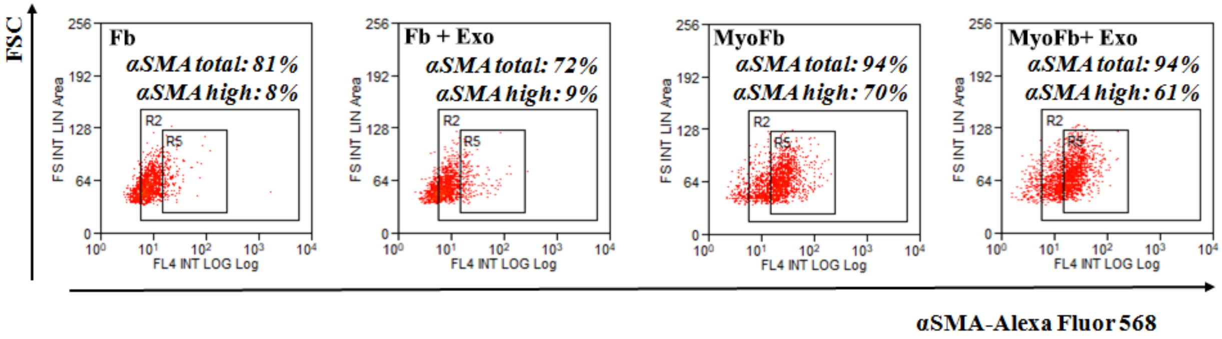

Figure S2. Flow cytometry analysis for the expression of αSMA on dermal fibroblasts stimulated with exosomes (Exo), TGFβ1 and TGFβ1+Exo

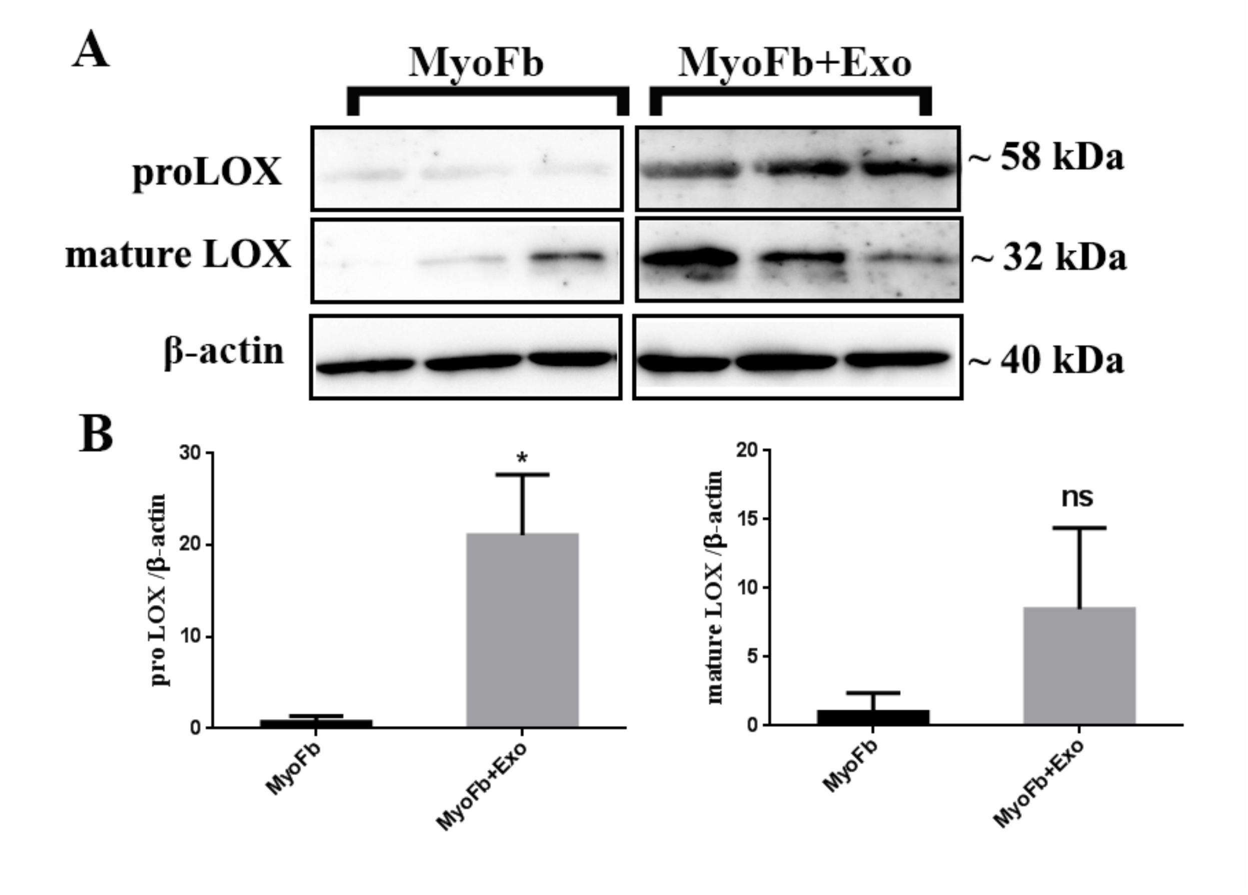

Figure S3. Western blot analysis for the expression of lysil oxidase (LOX)- proenzyme (proLOX) and mature forms, in dermal myofibroblasts and myofibroblasts treated with exosomes.

Table S1. List of primer sequences

**Gene**	**Primer sequence**
COL1A1	Forward: 5'- GTCACCCACCGACCAAGAAACC -3'<br>Reverse: 5'- AAGTCCAGGCTGTCCAGGGATG -3'
COL3A1	Forward: 5'- GGTGTCCCAGGGAAAGATGG -3'<br>Reverse: 5'- TATACCTGGAAGTCCGGGGG -3'
ACTA2	Forward: 5'- GGCAAGTGATCACCATCGGA -3'<br>Reverse: 5'- GTGGTTTCATGGATGCCAGC -3'
TIMP1	Forward: 5'- ACTGATGGTGGGTGGATGAG -3'<br>Reverse: 5'- ATGGTGGGTTCTCTGGTGTC -3'
TIMP2	Forward: 5'- TGAGAAGGAAGTGGACTCTGG -3'<br>Reverse: 5'- CCTTTCCTGCAATGAGATATTCC -3'
MMP2	Forward: 5'- ACTACAACTTCTTCCCTCGCA -3'<br>Reverse: 5'- GGCATCATCCACTGTCTCTG -3'
MMP13	Forward: 5'- GCAAACTTGACGATAACACCT -3'<br>Reverse: 5'- TCGCCATGCTCCTTAATTCC -3'
MMP14	Forward: 5'- TGAGGATCTGAATGGAAATGAC -3'<br>Reverse: 5'- GGGTTTATCAGGAACAGAAGG -3'
EMMPRIN	Forward: 5'- GCTACTGCCATCCAATCGAG -3'<br>Reverse: 5'- TCTTTCTTGGTCTGCATTCAC -3'
GAPDH	Forward: 5'- CGCTCTCTGCTCCCTCCTTGT -3'<br>Reverse: 5'- CCATGGTGTCTGAGCGATGT -3'
